# Supplementary material for: Are carbon emissions trading and green financial instruments synergistic? -Comprehensive quantitative research based on content analysis
Source: PLoS One. 2024 Mar 7;19(3):e0298601. doi: 10.1371/journal.pone.0298601 (PMC10919601; doi:10.1371/journal.pone.0298601)
Supplement: S1 Data — (ZIP) [file pone.0298601.s001.zip › Data/地级市Z_F工作报告.zip!/║■▒▒/╧σ╤⌠╩╨/└┤╫╘╣1⁄2╓┌║┼í╛┬φ┐╦╩2╛▌═°í┐_2019.docx]

各位代表：

现在，我代表市政府向大会报告工作，请予审议，并请政协委员和其他列席人员提出意见。

一、2018年工作回顾

过去的一年，面对稳中有变、变中有忧的新挑战，我们以习近平新时代中国特色社会主义思想为统领，认真贯彻落实党的十九大和习近平总书记视察湖北重要讲话精神，深入践行新发展理念，着力实施高质量发展十大重点工程，“一极两中心”建设迈出坚实步伐。

——经济运行稳中有进。主要经济指标持续回升，地区生产总值同比增长7.8%，规模以上工业增加值增长8.1%，固定资产投资增长11.5%，社会消费品零售总额增长11.9%，地方一般公共预算收入可比增长8%，外贸出口额增长26.7%，实际外商直接投资额可比增长16.5%，全面完成年初确定的目标任务，其中规模以上工业增加值、固定资产投资、社会消费品零售总额、外贸出口额、实际外商直接投资额增速均高于全省平均水平。综合实力进一步增强，经济总量全省第二的地位更加巩固，纳入全省考核的7个县（市、区）均被评为“全省县域经济工作成绩突出单位”。经济结构优化效益向好，预计地区生产总值中服务业占比达到38%以上，同比提高约3个百分点；工业增值税增幅高于全省平均水平，税收占地方一般公共预算收入的比重达到70%。

——新旧动能转换加快。产业转型升级成效明显，10个产业集群获得省考核认定，39个工业大类行业中34个实现增长，高技术制造业产值增长13.8%，新能源新材料、电子信息、农产品加工、装备制造等产业产值同比分别 增 长 32.4%、 14.1%、 12.2%和15.4%；我市汽车产业在全国汽车产销负增长的情况下逆势而进，整车产量38.4万辆、同比增长23.4%，其中新能源汽车产量突破4万辆、同比增长23.8%，国家智能网联汽车质量监督检验中心获批，东风汽车试验场四期扩建及智能网联汽车小镇开工建设，智能网联汽车道路测试工作正式启动。创新能力持续增强，新认定高新技术企业65家，规模以上高新技术产业增加值同比增长13.3%；新增国家级和省级研发创新平台9个、院士专家工作站20个，转化科技成果105项；高新区成为国家高端装备制造业（新能源汽车）标准化试点和国家知识产权示范园区，枣阳市、老河口市获批省级高新技术产业开发区。

——发展活力明显增强。要素集聚力持续提升，新引进世界500强、中国500强及上市公司37家，新引进亿元以上项目650个，其中有42个工业项目投资过10亿元，3个工业项目投资过50亿元。市场主体加快成长，新注册企业1.53万家，新增规模以上工业企业80家、限上商贸企业112家，骆驼集团、立晋钢铁等5家企业入围湖北民营企业100强，美亚达新型建材、龙蟒钛业等13家企业入围湖北民营企业制造业100强，葵花药业、新火炬等19家企业成为全省隐形冠军和科技小巨人企业，三环锻造获得“湖北省第七届长江质量奖”。

“双创”活力持续迸发，新增国家级和省级“双创”载体17个，市人才创新创业超市成为国家级中小企业公共服务示范平台，新引进53个高层次团队创新创业、718名高层次人才全职工作、2.25万名大学生就业创业，成功举办中国创新创业大赛湖北赛区总决赛和汉江创客英雄汇。

——绿色发展深入推进。减量化增长方式扎实推行，75个工业节能和清洁生产项目投入运行，东风康明斯被评为国家级绿色工厂，老河口市成为国家资源循环利用基地；化肥施用量亩均减少0.5公斤，化学农药使用量减少1%，我市成为全国畜禽粪污资源化利用整市推进试点；城市生活垃圾、餐厨垃圾、建筑垃圾无害化处理能力和资源化利用水平全省领先；新增绿色建筑750万平方米、装配式建筑40万平方米；预计单位生产总值能耗、二氧化碳排放分别下降4%和4.3%，化学需氧量、氨氮、二氧化硫、氮氧化物比“十二五”末分别下降8.2%、9.3%、25%和12.8%，全面完成省定目标。坚决整治突出环境问题，中央和省环保督察反馈问题年度整改任务全面完成。以前所未有的力度实施大气污染综合整治，在全年降雨量比上年减少三分之一（减少428毫米）的不利条件下，中心城区PM10平均浓度同比下降1.1%，PM2.5平均浓度同比下降7.6%，空气质量优良天数比例达到67.1%，完成省定考核目标。水环境质量持续改善，国家和省考核我市的汉江及其支流9个断面水质均值达标率100%，县级以上集中式饮用水水源地水质达标率100%，我市在全国水生态文明试点城市验收中获得优秀等次。生态建设成效显著，保康县成为国家生态文明建设示范县，新增3个省级森林城镇、125个省级绿色乡村。

——人民生活持续改善。年度脱贫攻坚任务全面完成，76个贫困村出列，76247人脱贫，南漳、保康、谷城三县“摘帽”正按计划接受中央和省考核验收。城镇新增就业10.4万人，社会养老保险待遇连续十四年提标，15家医院实现异地就医结算，城乡居民医保个人支出比例进一步降低，提供医疗救助13.6万人次、困难群众生活救助13.4万人，解决了32万农村居民饮水安全问题。城乡居民人均可支配收入增速高于地区生产总值增幅，物价保持总体稳定。扫黑除恶专项斗争取得阶段性胜利，打掉涉黑犯罪组织11个、涉恶犯罪集团和团伙132个，群众安全感进一步提升。

一年来，我们突出抓了以下八个方面的工作：

（一）全力振兴实体经济。狠抓政策落地提振企业信心，为企业减税降费83亿元，兑现惠企资金35亿元，减少电费支出2.94亿元。狠抓服务企业破解发展难题，帮助企业解决具体问题1000余个，为100多家企业提供了融资支持。狠抓招商引资和项目建设壮大投资支撑，“楚商襄阳行”、承接大湾区产业转移专题推介等17场专题招商活动成果丰硕，全年实际到位资金2800亿元；强化项目拉练等推进机制，项目建设提速增效，江山重工军民融合高端装备、双林汽车轴承、啸创汽车配套加工基地等193个亿元以上项目开工建设，天药药业、正大熟食、唐城夜游等117个亿元以上项目竣工，全省投资和重大项目谋划推进工作现场会在我市召开。

（二）全力推进产业提质升级。扎实开展工业高质量发展行动，实施亿元以上技改项目95个，技改投资同比增长43.1%，新增智能工厂和机器换人示范企业30家、数字化生产线105条；实施亿元以上新兴产业项目117个，汇尔杰玄武岩纤维等75个项目竣工投产。着力补强服务业短板，新增28家国家A级和省级重点物流企业，创建3个省级电子商务示范基地，电子商务交易额同比增长20%，我市成为全省服务外包示范城市；襄阳华侨城文化旅游示范区等重大文化旅游项目开工建设，九路寨、香水河成功创建4A级景区，全市A级以上景区接待游客1202.3万人次，实现门票收入3.35亿元，同比分别增长35.7%和29.2%，我市成为全国旅游标准化试点城市。

（三）全力推进乡村振兴。优化农业供给体系，新建高标准农田105万亩、“稻虾共作”基地12万亩，新建改造茶园5万亩，新认证“三品一标”农产品82个，新注册地理标志商标13件，新增市级以上示范合作社和家庭农场120家，以襄州为核心区的襄阳国家农业科技园区获批，南漳县成为全国农村一二三产业融合发展先导区，枣阳市、宜城市、南漳县被评为全国主要农作物全面全程机械化示范县（市）。扎实推进“百村示范、千村推进”和农村清洁工程，深入开展“农村清洁家园百日行动”，建成26个省级美丽乡村示范点，90%以上乡村生活垃圾实现集中收集处理。农村集体产权制度改革走在全省前列，宜城市农村土地制度改革三项试点经验在全国推广。

（四）全力打好三大攻坚战。在防范化解重大风险攻坚上综合施策，全年化解不良贷款82.8亿元，非法集资存案和新发案件实现“双降”，政府债务额度控制在合理区间；社会治理力度加大，社会治安、安全生产和食品药品安全形势稳定，全年未发生较大以上安全生产事故，安定和谐的局面持续巩固。在脱贫攻坚上精准施策，“扶贫车间”、“合作社+农户”、电商扶贫等一批产业扶贫模式有效推广，2.1万个贫困户获得小额扶贫贷款支持，专业合作社覆盖到所有贫困村；完成易地扶贫搬迁8877户，改造贫困户危房9899户；建档立卡贫困人口实现基本医疗保险、补充医疗保险全覆盖，住院实际报销比例提高到90%以上；为11.2万名建档立卡贫困家庭学生提供了教育资助。在污染防治攻坚上重拳出击，深入开展蓝天、碧水、净土行动，中心城区空气质量持续好转，汉江生态环境问题大排查大整改取得积极成效，26家危化品企业及沿江化工企业搬迁全面启动，取缔非法采砂场396个、拆除非法船舶1086艘，“厕所革命”、精准灭荒年度任务全面完成，全市农田土壤环境质量均达到清洁等级。

（五）全力提升城市功能品质。中心城区改造棚户区2万套，推进建设棚改安置房1.4万套，综合整治背街小巷165条，旧城面貌进一步改观；东西轴线全线开工，苏岭山大桥即将通车，打通“断头路”、增设停车位、完善公交体系等取得新成效，交通功能进一步提升；新建改建一批标准化菜市场、公共厕所、垃圾转运站，综合整治积水渍涝点，积极推行生活垃圾分类管理，市政功能进一步提升；鱼梁洲环岛景观带、樊城江滩公园二期、滨江路东延伸段景观公园等工程快速推进，襄新大道绿化工程全面完工，新增绿地262万平方米，生态品质进一步提升；全国文明城市创建纵深推进，城市管理精细化水平明显提升，清洁家园活动成效显著，铁腕拆违有力遏制了违法建设，城市更加干净有序。加快建设全国性综合交通枢纽，汉十高铁、郑万高铁、蒙华铁路襄阳段进入铺轨阶段，配套工程同步实施，东津高铁站承轨层及地下主体工程完工；襄阳机场新开通航线6条、通航城市达到27个，旅客吞吐量突破140万人次、同比增长36.9%；汉江雅口枢纽投资与形象进度“双过半”，小河港区一期工程水工建筑基本完工；枣阳至潜江等5条高速公路建设进展顺利，新建一二级公路157公里、农村公路1020公里，提档升级农村公路1513公里，南漳县、老河口市成为全省“四好农村路”示范县。

（六）全力深化改革开放。放管服改革持续突破，市县两级政务服务“一张网”全面建成运行，196项市级行政审批事项实现“最多跑一次”，“证照分离”改革将企业开办时间压缩至3个工作日以内，工业项目“先建后验”的做法得到李克强总理批示肯定，“大幅简化退税流程”做法获得国务院表彰。财政和投融资体制改革持续深化，预算绩效管理全面实施；新落地PPP项目13个、总投资156.9亿元，入选财政部PPP示范项目8个、数量居全省第一；发行各类债券75.4亿元，226家企业在武汉股权托管交易中心挂牌。国企国资改革进一步深化，汉江国投转型发展步伐加快，国有企业职工家属区“三供一业”分离移交基本完成。开放发展取得新成效，襄阳自贸片区综合保税区起步区启动建设，国际贸易“单一窗口”企业覆盖率达到90%以上，新落户项目48个、总投资221.5亿元；襄阳至宁波港国际铁海联运、“襄汉欧”“襄渝欧”中欧货运班列实现常态化运营。

（七）全力增进人民福祉。年初确定的民生实事35个子项中，有33项全面完成；未按时全面完成的2项是，人行天桥建设项目（计划增设16座人行天桥，主体工程已完工，正在建设配套设施，春节前可投入使用）、建制镇污水处理设施项目（计划建成78个，目前6个未建成，预计1月底前5个可建成，4月底前最后一个可完工）。就业服务进一步加强，为894家用工企业发放稳岗补贴3557.8万元，为创业载体和创业主体发放奖补资金1225.6万元。社会保障力度加大，机关事业单位养老保险制度改革进一步深化，困难职工养老保险“助保贷”启动实施，城乡居民医保制度进一步完善，“五险”扩面新增30.25万人次。教育事业实现新发展，公益普惠性学前教育办学规模进一步扩大，义务教育薄弱学校改造力度加大，襄阳三中新校区投入使用，市三十六中迁建工程基本完成，襄阳四中新校区建设快速推进，湖北文理学院成为硕士学位授予单位。公共文化服务体系进一步完善，市图书馆主体竣工，市博物馆开工建设，襄阳古城墙、小江西会馆等文物修缮项目完工，改扩建乡镇综合文化服务中心18个，931个试点村（居）文化小康建设扎实推进，成功举办诸葛亮文化旅游节、襄阳马拉松等赛事，长渠（白起渠）成功申遗。卫生健康工作得到加强，基层医疗卫生服务功能进一步提升，公立医院综合改革在全省考评中名列市州第二，药品带量采购实现二级以上公立医院全覆盖。

（八）全力提升政府效能。树牢“四个意识”、践行“两个维护”，不折不扣落实党中央决策和省委省政府部署，始终做到市委凡有决定、政府必有行动、行动必有效果。以“落实年”和“打造新时代襄阳产业文化大讨论”活动为契机，狠抓转作风提效能，市政府领导成员既当指挥员又当战斗员，对市委市政府确定的重点工作人人领衔主抓、分兵把口落实，坚持“一线工作法”现场办公解决问题、“三不一直”调研督办推动工作；对发展中的大事快议快定快干，比如针对服务业短板突出、贷款不良率上升、营商环境不优、政府投资项目管理不严等，迅速研究推出有效措施；对工作中的难事咬住不放钉钉子，比如针对中央环保督察反馈问题整改、大气污染治理、棚户区改造、河道非法采砂和非法码头整治等硬骨头，重拳出击逐个攻坚；对市场主体的急事一一回应快速处理，比如针对招商合同兑现难、中小企业续贷周转难、部分重点企业遭遇资本市场风险等问题，果断出手逐个破解；对久拖未决的突出矛盾敢于担当积极化解，比如针对中心城区37个征迁安置房建设项目延期交房问题，逐一研究、逐个制定有效解决方案并扎实有力推进，目前已分配入住9097户。我们坚持用法治思维与法治方式开展政府工作，自觉接受市人大及其常委会法律监督、工作监督和市政协民主监督，积极回应社会和舆论关切，认真办理人大代表建议和政协委员提案。

与此同时，国防动员、“双拥”共建、对口支援等工作继续加强，慈善、助残、妇女儿童、红十字事业等都实现了新发展，审计、统计、气象、档案、保密、史志、对台、外事侨务、民族宗教、机构编制等方面的工作都取得了新成绩。

各位代表！过去一年，尽管我们遇到的困难前所未有，但取得的成绩好于预期。这主要得益于习近平新时代中国特色社会主义思想的科学指引，得益于省委省政府和市委的坚强领导，得益于全市各级干部群众的奋力打拼。在这里，我代表市政府，向全市人民，向各位人大代表、政协委员，向各民主党派、各人民团体和社会各界人士，向驻襄人民解放军、武警官兵和政法干警，向所有在襄投资者、创业者、建设者，致以崇高的敬意，表示衷心的感谢！

我们也清醒地看到，全市经济社会发展不平衡不充分的问题还比较突出。经济下行压力加大，实体经济、民营经济困难增多，营商环境还需进一步优化；新旧动能转换任务艰巨，传统产业调整转型阵痛加剧、新兴产业支撑力不足、服务业占地区生产总值比重低于全国全省平均水平、农业供给质量不高、科技创新能力不强等问题依然突出；生态环境保护任务艰巨，特别是大气污染防治形势依然严峻，农业面源污染、水环境污染等问题比较突出；优质公共服务供给不足，教育、医疗、住房等领域短板较多；少数公务人员懒政、滥政、粗放施政的现象还比较突出，政府工作效能有待进一步提高，等等。对此，我们将采取有效措施，切实加以解决。

二、2019年重点工作

2019年是新中国成立70周年，是决胜全面建成小康社会的关键之年。当前，我们面临着外部环境趋紧但内部环境宽松的新形势，既有经济运行稳中有变、实体经济困难加剧的严峻挑战，也处于经济基本面稳中向好、多重利好叠加释放的重要战略机遇期。尤其是国家强化逆周期调节，出台实施“六稳”举措、促进形成国内强大市场等政策，开启了重要政策窗口期；国务院正式批准实施《汉江生态经济带发展规划》，明确了襄阳汉江流域中心城市的战略地位，蕴含着难得的发展机遇；省委省政府作出打造“一芯驱动、两带支撑、三区协同”的高质量发展区域和产业战略布局重大部署，进一步提升了襄阳在全省区域发展格局和产业战略布局中的支撑地位，赋予了襄阳引领区域高质量发展的重大使命，为我市注入了新的强劲发展动能；全国性综合交通枢纽加速形成，襄阳即将进入高铁时代，要素吸纳集聚和区域辐射带动功能显著提升；近年来我市改革创新持续突破，高质量发展十大工程扎实推进，减量化增长深入实施，一批重大项目相继引进、建成投产，发展后劲不断增强。只要我们保持战略定力、坚定发展信心，紧紧抓住用好这些重大机遇和有利条件，一定能够把握发展主动权，开创发展新局面。

今年政府工作的总体要求是：以习近平新时代中国特色社会主义思想为指导，全面贯彻党的十九大和十九届二中、三中全会精神，坚决落实习近平总书记视察湖北重要讲话精神，统筹推进“五位一体”总体布局，协调推进“四个全面”战略布局，坚持稳中求进工作总基调，深入践行新发展理念，以深化供给侧结构性改革为主线，贯彻巩固、增强、提升、畅通的方针，工程化项目化推进高质量发展，加快建设现代化经济体系；以推进减量化增长为抓手，促进产业转型升级、发展方式转变，积极创造绿色发展新示范；以深化改革开放为动力，提升创新力，增强竞争力，充分释放体制机制活力，更好发挥汉江生态经济带战略引擎作用；以决胜全面建成小康社会为目标，落实“六稳”工作部署，坚决打好“三大攻坚战”，提高人民群众获得感、幸福感、安全感，确保经济发展质效、城市发展能级、乡村振兴实效、民生改善幅度、社会治理水平走在全省前列、领先汉江流域，为全面建设社会主义现代化强省作出新贡献。

今年的主要预期目标是：地区生产总值增长8%左右，规模以上工业增加值增长8%，固定资产投资增长11%，社会消费品零售总额增长11%以上，地方一般公共预算收入可比增长8%，外贸出口和实际外商直接投资额分别增长8%，城镇登记失业率控制在4.5%以内，城乡居民人均可支配收入增长与经济增长同步，全面完成防范化解重大风险、精准脱贫、污染防治、节能减排、安全生产等省定目标。

新的一年，我们将扎实抓好以下八个方面的重点工作：

（一）推动经济稳中有进

提高投资质量效益。聚力精准招商，搭建专业化招商平台，探索市场化招商机制，以承接大湾区、长三角、京津冀等地区产业转移为重点，推动产业链招商实现新突破，力争新引进投资过5亿元的工业项目120个、过亿元的新兴产业项目100个、现代服务业项目120个，新引进世界500强、中国500强及上市公司35家以上，招商引资实际到位资金突破3000亿元。推动项目建设提质提速，抢抓政策窗口期机遇，重点对接长江经济带、汉江生态经济带等战略规划，努力在争政策、争项目、争资金上实现新突破。完善项目拉练和“三集中”机制，抓好卡为智能终端产业园、骆驼动力电池梯次利用及再生产业园、中车交通汉江捷运装备总部基地等投资过50亿元的工业项目，加快推进金环绿色纤维素纤维、航宇嘉泰航空座椅、高德红外等16个投资过10亿元的工业项目，确保竣工投产省市重点项目20个以上、过亿元工业项目130个以上，壮大高质量发展硬支撑。

促进消费提质升级。推动中心城区打造集购物、娱乐、餐饮、体验等服务功能为一体的特色商业街区，支持大型商超引进国际名企、名店、名品入驻，支持樊西现代服务业集聚区引进大型商贸企业和先进商贸业态，着力打造区域性消费中心。开展社区生活综合服务中心建设试点，积极引进新零售模式，促进社区生活服务业集聚发展。积极倡导绿色消费，培育网络、定制、体验、智能、时尚等消费新热点。加快构建农村现代流通体系，推进电子商务进农村综合示范建设，促进农村居民消费升级。

支持民营企业发展。完善市级领导定期走访联系民营企业制度，建立民营企业诉求收集、交办、催办、办结反馈“直通车”机制，千方百计帮助重点民营企业纾困，“一企一策”支持骨干企业发展，加快处置“僵尸”企业。下大力气缓解融资难融资贵，组建政府性融资担保集团，建立政银担风险分担机制，鼓励金融机构开展应收账款融资、“银税互动”等金融产品创新，力争在推进民营企业上市和债券融资上实现新突破。完善政策执行方式，推动各项惠企政策措施落地落细落实，切实减税降费，降低物流、用能等成本，实质性减轻企业负担。全面实施市场准入负面清单制度，破除民间资本进入的隐性障碍。切实保护企业家合法的人身和财产权益，保障企业合法经营。开展营商环境评价和突出问题治理，构建亲清新型政商关系，真正把企业的事当成自家事，把企业家当成自家人，让广大投资者实实在在感受到投资襄阳更安全、更公平、更便利。

（二）培育壮大经济新动能

加快改造升级传统产业。重点抓好260个投资过千万元的技改项目，力争170个竣工投产，建设智能工厂10个、机器换人示范企业30家、数字化车间27个、数字化生产线130条，创建省级服务型制造示范企业5家以上。力争新增规模以上工业企业80家，新增省级专精特新“小巨人”和制造业单项冠军20家以上。推动十大省级成长型产业集群转型升级，支持东风公司襄阳基地导入新车型、建设新项目，促进新天籁、奇骏和江南汽车产能释放，带动汽车零部件企业近地化配套。实施特色产业园区功能升级行动，力争10个特色产业园区实现“七通一平”，加快推进东风铸造产业园和宝武特钢产业园落地。

加快培育壮大新经济。着力壮大新产业，力争竣工投产亿元以上新兴产业项目33个；坚定不移推进“新能源汽车之都”建设，支持东风旅行车电动汽车产能释放，推动东风旅行车氢燃料电池客车、江南电动汽车实现量产，加快东风和中克骆瑞电驱动系统项目建设；积极发展智能网联汽车产业，加快建设东风汽车试验场四期、智能网联汽车小镇和智能网联汽车运营示范线。着力培育新业态，推动互联网、大数据、人工智能与实体经济深度融合，大力开展“千企登云”行动，加快建设国家工业互联网标识二级节点，在数字经济、智慧经济、分享经济、创意经济等新经济领域引进和孵化一批市场主体，积极创建国家级云计算产业示范基地和软件服务外包基地，力争襄阳高新区新经济指数居全国同类高新区前列。深化创建国家军民融合创新示范区，实施国防科技工业军民融合深度发展、军民科技协同创新转化、基础设施军地共建、军队保障社会化推进、紧急动员力量融合等五大工程，加快建设12个军民融合重点产业项目，培育新的经济增长点。

加快发展现代服务业。推进服务业提速升级，力争服务业增加值占地区生产总值比重提高3个百分点、达到41%。突破性发展现代物流业，加快建设襄阳金鹰重工公铁联运物流园二期、襄阳传化物流港、京东亚洲壹号配送中心等重点物流项目，大力引进和培育骨干物流企业，抓好全国绿色货运配送示范城市和供应链创新与应用试点城市建设，支持樊城创建国家级现代商贸物流示范区，全力打造生产服务型国家物流枢纽城市。突破性发展文化旅游业，持续推进全域旅游发展，突出抓好襄阳华侨城文化旅游示范区、唐城三朝时空文化旅游区、古隆中三国文化主题营地等27个重点文化旅游项目，支持古隆中、尧治河创建国家5A级景区，力争创建4A级景区取得新成果，打造一批乡村旅游示范点，提升旅游供给质量；以全国旅游标准化试点城市建设为抓手，完善旅游公共服务设施体系，提升智慧旅游服务水平；抢抓高铁时代来临的重大机遇，大力实施“引客入襄”工程，扩大“襄阳好风日”旅游品牌的市场影响力，力争全年A级以上景区接待游客、门票收入分别比上年增长25%和20%以上。突破性发展健康养老服务业，全面放开养老服务市场，鼓励社会力量兴办养老和医疗健康服务机构，发展医养结合模式，培育健康养老服务新业态。

加快推进创新发展。全力推进减量化增长，建立健全长效投入和考核评价机制，推动高质量发展十大重点工程深入实施，加快推进工业“四化四造”、服务业信息化改造和农业循环发展，打造一批清洁生产、绿色制造示范企业和循环农业试点示范。大力实施科技创新高质量发展三年行动计划，加快建设综合型产业技术研究院和智慧化科技资源共享服务平台，积极引进研发检测、工业设计等科技服务企业集聚发展；支持企业加强产学研合作，创建国家和省级研发平台；完善科技成果转化体系，转化科技成果100项以上；加快孵化培育科技中小企业，新认定高新技术企业65家以上；积极创建国家知识产权示范市，支持宜城市、谷城县创建省级高新技术产业开发区。打造“隆中对·双创”升级版，实施“隆中人才振兴工程”，重点支持科研人员和大学生创业创新，建成襄阳科技城二期，加快建设国际人才港和外国专家创新示范基地，完善人才住房保障体系，力争引进高层次人才创新创业团队60个、全职工作高层次人才750名、创业就业大学生2万名以上；完善人才政策，培养留住本土人才。实施技能强市工程，引进和培养一批高技能人才。

（三）深入实施乡村振兴战略

深化农业供给侧结构性改革。大力发展特色优势农业、培育特色农产品品牌，提升“中国有机谷”建设水平，有机农产品认证基地达到25万亩，新建“稻虾共作”基地10万亩，改扩建茶园3万亩，新增“三品一标”农产品40个。加快推进一二三产业融合发展，大力引进和培育农产品精深加工龙头企业，推动建立“公司+基地+农户”的利益联结机制，建设100个休闲农业示范点。加强农业生产能力建设，加快实施鄂北水资源配置工程襄阳分水口建设，抓好大中型灌区续建配套与节水改造，完成124座小型水库除险加固，积极推进高标准农田建设和襄北地区“旱改水”，稳定百亿斤粮食产能；扎实抓好国家和省级农业科技园区建设，提升主要农作物耕种收综合机械化水平。

建设美丽宜居乡村。扎实开展“百村示范、千村推进”三年行动，持续推进10条示范线建设；扎实抓好农村清洁工程，按照“码整齐、扫干净、清塘沟、保常态”的要求，持续开展农村人居环境整治；认真落实《襄阳市农村生活垃圾治理条例》，完善农村生活垃圾收集、清运、处置体系，推进农村生活垃圾治理全覆盖；开展“厕所革命”攻坚行动，探索建立乡村公厕长效管护机制；全市乡镇污水处理厂（站）全面投入运行，实现乡镇污水治理全覆盖，探索联户、整村生态净化处理模式。加强农村面源污染治理。实施村容村貌提升工程，分类推进农村房屋立面改造、村组道路提升，因地制宜建设“小三园”，让小花园、小果园、小菜园成为美丽乡村的亮丽风景。

创新“三农”发展机制。深入实施“三乡”工程，推动城市资金、技术、人才、信息等要素向农村集聚。壮大新型农业经营主体，新增市级以上示范合作社、家庭农场100家；培育新型职业农民4700人，鼓励利用当地优势创业创新，发挥其在乡村振兴中的主体作用；完善社会化服务体系，提高农业社会化服务水平。持续推进农村承包地“三权分置”改革，探索完善经营权抵押担保机制，深化农村集体产权制度改革，稳步推进农村金融合作试点工作，激发农业农村发展动能。扎实做好村级发展规划，加快构建自治、法治、德治相结合的现代乡村治理体系，让农村充满活力、和谐有序。

（四）坚决打好三大攻坚战

确保不发生系统性风险。规范政府举债融资行为，落实政府债务预算管理和限额管理政策，加快平台公司市场化转型，依法防范和处置地方政府隐性债务风险。认真落实支持金融机构化解不良贷款十项措施，扎实开展非法集资、非法校园贷、互联网金融违法违规行为等专项整治，严密防控企业债券兑付风险和重点企业债务偿还风险。统筹做好社会风险防范化解工作。

确保基本消除绝对贫困。巩固拓展精准脱贫成果，整合使用各类涉农资金，加大扶贫投入力度；突出抓好产业扶贫，统筹落实健康、就业、教育、文化等扶贫措施，增强贫困地区“造血”功能；坚持扶贫与扶志、扶能、扶智相结合，激发贫困群众内生动力。严格按照“两不愁、三保障、一高于一接近”的标准，抓紧补齐各项短板，以实实在在的脱贫成果迎接中央和省考核验收，确保南漳、保康、谷城三县顺利实现“摘帽”。

确保环境质量持续改善。以强烈的政治意识抓好中央和省环保督察反馈问题整改，全力解决突出环境问题。以更大力度打赢蓝天保卫战，坚持市县联动、全民参与、科学施策、标本兼治，推动重点行业实施特别排放限值、开展超低排放改造，全面开展工业窑炉、挥发性有机物、工业企业无组织排放、“散乱污”企业、建筑工地和道路扬尘、汽车尾气等专项整治行动，力争空气质量优良天数比例达到70%。坚决落实“湖北长江大保护十大标志性战役”总体部署，全面开展汉江大保护专项行动，着力消除水环境风险，确保省考核的9个断面水质全部达标。扎实开展净土行动，抓好土壤污染治理与修复。积极推进国家生态文明示范市和省级生态县乡村四级联创。持续开展“绿满襄阳”提升行动，扎实抓好精准灭荒、鄂北生态防护、汉江沿线生态修复等重点工程。完善环境保护长效机制，开展环境保护综合执法行动，压紧压实河湖长和山林长责任，努力把湖北重要生态屏障筑得更牢固。

（五）加快提升城市能级

提升中心城市功能。科学编制新一轮城市总体规划，做好总体城市设计，推动落实“多规合一”。加快城市新区建设，大力推进东津中心商务区、高铁站区等重点区域开发，启动建设未来科技城、博览城、东津新镇，加快建设大数据产业园、大湾区智能装备产业园，形成产城融合发展新格局；启动建设鱼梁洲中央生态公园，建成环岛景观带，提升城市“绿心”功能；加快建设庞公新区，推动中德生态示范城建设，完善樊西功能区承载功能，优化城市格局。推进旧城更新提质，用两年时间基本完成中心城区棚改项目，让棚户区居民尽快“出棚进楼”；继续抓好老旧小区和背街小巷综合整治，让更多市民住的舒心。推进路网体系建设，积极谋划推进城市轨道交通项目，加快建设东西轴线，开工建设南北轴线南延段，实施中环东线建设和内环快速化改造工程，确保庞公大桥主体完工；积极创建“公交都市”，完善公共交通体系，让市民出行更便捷。推进市政功能提升，加强停车位、菜市场、公共厕所、垃圾转运站等市政设施配套，抓好供热、供气、供水、供电等设施和管网建设，继续实施城区供暖工程，加快实施城市排涝能力提升和污水处理提质增效行动，让市民生活更便利。推进城市品质提升，启动古城人口和功能疏解，实施古城街景整治、陈老巷修复、管家巷改造提升等工程，再现襄阳古城历史风貌；抓好城市道路景观、公园游园、城市绿道、拆墙透绿和庭院美化等绿化美化工程，让城市颜值更高、气质更好。坚持全域全民推进全国文明城市创建，认真落实《襄阳市文明行为促进条例》，持续开展不文明行为专项整治，扎实办好创建惠民实事，打造一批文明示范创建点，让人民群众在创建中得到更多的实惠；深化城管体制改革，提升城市管理科学化、精细化水平，落实《襄阳市城市生活垃圾治理条例》，让市容更干净、秩序更井然。

提升综合交通枢纽功能。全力建成蒙华铁路、汉十高铁襄阳段、郑万高铁襄阳北段和东津高铁站，加快推进呼南高铁襄阳段落地建设，积极争取合西高铁过境襄阳。加快实施襄阳机场改扩建工程，促进通用航空产业发展。加快实施绕城南、枣阳至潜江北段、保康至神农架等高速公路续建工程，确保枣阳至潜江南段高速公路建成通车，开工建设襄阳至南漳高速公路；加快建设河谷大桥、宜城汉江二桥等跨江节点工程，启动207国道绕城改线。开工建设汉江新集枢纽，实现雅口枢纽蓄水通航，力争完成小河港区一期陆域工程。加快构建多式联运的现代综合运输体系，提升“七省通衢”的区位优势。

提升区域协调发展水平。抢抓汉江生态经济带发展规划实施的重大机遇，争取启动实施一批基础性、支撑性项目，探索建立流域合作共建机制。全面落实全省高质量发展区域和产业战略布局新部署，在“一芯两带三区”中找准位置，在提升省域副中心发展能级上全力突破，在建设综合性国家产业创新中心、“芯”产业智能制造中心、制造业强国示范中心上积极作为，着力打造汉随襄十制造业高质量发展带的重要引擎、鄂西绿色发展示范区的重要增长极、江汉平原振兴发展示范区的重要支撑。推动城区经济和县域经济高质量特色化发展，力争襄阳高新区创新实力在全国高新区中位次前移，支持东津新区打造国家产城融合示范区，支持鱼梁洲创建国家旅游度假区，支持纳入省考核的7个县（市、区）在全省县域经济比拼中争先进位。推动小城镇提升承载功能，因地制宜培育特色小镇，增强产业和人口集聚能力，加快形成县域特色产业增长极。

（六）持续深化改革开放

深化“放管服”改革。启用汉江北路新市民服务中心，全面推进行政审批“三集中三到位”，实现市县乡村政务服务“一张网”全覆盖，“最多跑一次”比例达到90%以上。全面推行“证照分离”，实现“持照即可经营”；全面深化“多证合一”改革，扩大“一照一码”范围；开展“多评合一、多审合一、多验合一”，完善工业项目先建后验，开展项目报建审批区域性统一评价，实现“拿地即开工”。创新市场监管方式，完善“双随机一公开”机制；健全社会信用体系，加大守信激励和失信惩戒力度。

推进重点领域改革。按照省委省政府和市委统一部署，全面完成政府机构改革各项任务。稳妥推进事业单位分类改革，继续推进生产经营类事业单位转企改制。深化综合执法体制改革，统筹配置行政处罚职能和执法资源，组建市场监管、生态环境、文化市场、交通运输、农业等领域综合执法队伍。建立全面规范透明、标准科学、约束有力的预算制度，加快实现预算和绩效管理一体化；优化财政支出结构，牢固树立过紧日子的思想，大力压减一般性支出，把更多资金用于脱贫攻坚、民生保障、“三农”发展、结构调整、科技创新和生态环保等领域；推进政府购买服务机制改革，完善政府采购制度和国库集中支付制度。坚持以管资本为主加强国有资产监管，积极稳妥推进混合所有制改革，加强现代企业制度建设，支持汉江国投做优做强，提升国有经济发展质量和效益。

推进高水平开放。加快襄阳自贸片区建设，用好用足下放的国家和省级经济社会管理权限，探索形成更多可复制推广的制度创新经验。加快建设开放平台，积极申报一类航空口岸和综合保税区，确保综合保税区起步区、自贸片区综合服务中心全面建成，大力推广应用国际贸易“单一窗口”。加快开放大通道建设，强化铁路枢纽功能，提升铁路运输占比；扩大襄阳至宁波铁海联运、“襄汉欧”国际货运班列运营成果，拓展国际物流新通道。加快发展开放型经济，加强与“一带一路”沿线国家和地区合作，推动产能、技术、产品“走出去”；加快培育优势产品出口集群，力争新引进1-2家外贸综合服务企业；提升利用外资质量，扩大利用外资规模。

（七）着力保障和改善民生

提高就业和社会保障水平。扎实抓好就业这个最大的民生，加大企业稳岗和职工培训支持力度，抓好下岗失业人员培训，及时开展下岗失业人员帮扶，促进就业稳定；全面落实支持大学生、退役军人和返乡人员就业创业政策措施，城镇新增就业7.5万人。深入推进“全民参保计划”，扎实做好中小企业、灵活就业人员等重点人群参保工作，深化机关事业单位养老保险制度改革，“五险”扩面新增16万人次，不遗余力兜牢社会保障这条底线。构建房地产市场健康发展长效机制，完善住房市场体系和住房保障体系。扎实开展社会救助，社会救助标准、补助水平均提高5%以上，医疗救助、临时救助年人均水平提高10%以上，失能、半失能农村五保对象集中供养率达到50%以上，加强对残疾人、留守儿童等特殊群体的关爱和帮助。

努力办好公平优质教育。继续实施学前教育三年行动计划，扩大普惠性学前教育规模。推进义务教育均衡发展，全面加强乡村学校、乡镇寄宿制学校和中心城区中小学校建设，加强教师队伍建设，保障义务教育教师工资待遇，加快消除“大班额”。扩大优质高中资源供给，建成襄阳四中新校区。积极发展特殊教育。深化产教融合，提高职业教育发展水平。提升高校办学水平，支持襄阳职业技术学院建设国家“双高”院校，启动湖北文理学院新校区建设。

加强文化小康建设。加快构建以市县乡（镇）“三馆一站”为主体的公共文化服务体系，加快建设市博物馆、科技馆、文化艺术中心、游泳健身中心，确保市图书馆和档案馆投入使用，开展新时代文明实践中心和县级融媒体中心试点建设。实施文化惠民工程，深入开展新时代“红色文艺轻骑兵”主题活动。加大文物保护修缮和非物质文化遗产保护传承力度。继续办好诸葛亮文化旅游节、襄阳马拉松、中国大学生方程式汽车赛等重大文体活动。

加快建设健康襄阳。深化“三医联动”改革，规范做实医联体建设和家庭医生签约服务，建立药品带量采购长效机制，实现总额预付、单病种付费等改革全域覆盖，着力解决看病难看病贵；加快推进市中心医院和市中医院东津院区建设，启动市疾控中心、市急救中心、市儿童医院迁建工程，提高乡镇卫生院、社区卫生服务中心医疗设备水平，加大基层医疗机构全科医生培养力度，完善精神障碍预防、治疗、康复体系和救助保障体系，努力为人民群众提供更高质量的医疗健康服务；推进电子健康卡与社会保障卡功能融合，提升群众就医便利化水平。推动计划生育服务管理转型提质，完善鼓励二孩生育政策，促进人口均衡发展。

着力提升社会治理水平。学习推广“枫桥经验”，创新群众工作方法，坚持推进依法治理，努力把矛盾化解在基层；全力打造“平安襄阳”升级版，深入开展提升群众安全感“六大行动”，实施网格化管理提升工程，健全完善立体化社会治安防控体系，纵深推进扫黑除恶专项斗争，为新中国成立70周年营造安定团结、和谐稳定的社会环境。建立健全应急管理体制机制，全面落实安全生产责任制，扎实开展隐患排查治理，坚决守住安全生产“红线”。深入开展“双安双创”行动，巩固提升国家食品安全城市创建成果，严厉打击保健品非法直销，让人民群众吃得更放心。深入推进“双拥”共建，做好退役军人服务工作。加强民族宗教工作，扎实做好对口支援工作。继续抓好第四次全国经济普查工作。

（八）切实加强政府自身建设

坚定政治站位。深学笃用习近平新时代中国特色社会主义思想，坚决落实新时代党的建设总要求，增强“四个意识”，坚定“四个自信”，坚决做到“两个维护”。全面加强政府系统政治建设，把党的全面领导贯彻到政府工作各领域各方面，对中央决策、省委省政府部署和市委决定，始终做到坚决贯彻不走样、忠实执行不打折、狠抓落实不懈怠。严肃政府系统党内政治生活，持续推进“两学一做”学习教育常态化制度化，深入开展“不忘初心、牢记使命”主题教育，以良好的政治生态汇聚推进“一极两中心”建设的强大合力。

坚持依法行政。牢固树立社会主义法治信仰，坚持用法治思维与法治方式谋划推动改革发展。坚持科学民主依法决策，全面推进政务公开，不断提高政府工作透明度。完善依法行政制度体系，加强和改进政府立法工作，健全完善政府法律顾问制度，坚持严格规范公正文明执法。加强诚信政府建设，建立健全“政府承诺+社会监督+失信问责”机制，开展“新官不理旧账”问题专项治理，以政府诚信引领社会诚信。依法接受人大法律监督和工作监督，自觉接受政协民主监督，主动接受社会监督和舆论监督，认真听取人大代表、政协委员、民主党派、工商联、无党派人士和各人民团体的意见建议，在监督中不断改进政府工作。

坚持务实担当。面对稳中有变的新形势，坚持解放思想敢担当，敢于变中求新、变中求进、变中突破，积极探索改革发展新举措；面对新时代发展新要求，坚持提升本领善担当，持续开展大学习、大调研，着力培养“五种思维”“八种本领”，善于将党中央方针政策和省委省政府工作部署与襄阳实际相结合，创造性开展工作。面对突出矛盾和问题，坚持动真碰硬勇担当，敢于啃硬骨头、挑重担子、接烫手山芋，千方百计化解难题促发展；面对改革发展稳定的艰巨任务，坚持狠抓落实强担当，完善人人钉钉子、事事马上办、件件落得实的工作机制，以真抓实干的作风提升工作效能。

坚持从严治政。严格履行市政府党组全面从严治党主体责任，全面加强政府系统党风廉政建设。坚决落实中央八项规定及实施细则精神，驰而不息整治形式主义、官僚主义，让干部从无谓的事务中解脱出来，集中精力干事业。坚决支持政治巡察、纪检监察和审计监督工作，严肃查处侵害群众利益的不正之风和腐败行为，着力打造清廉政府。

三、扎实办好民生实事

始终坚持以人民为中心的发展思想，紧扣人民群众最关心最直接最现实的利益问题，集中力量办好一批民生实事。

1.实现全市农村现行标准下贫困人口和贫困村全部脱贫。

2.实施农村饮水安全巩固提升工程，全面解决农村居民饮水安全问题。

3.新建改建农村公路1000公里，完成公路安全生命防护工程1000公里，加固维修危桥、涵洞20座。

4.新建改造农户无害化厕所15.5万座、农村公厕1097座、乡镇公厕113座、市区公厕183座、交通厕所10座、旅游厕所50座。

5.新建改建扩建普惠性幼儿园18所，新增学位3220个；开工建设中心城区中小学校12所（10所主体工程完工），新增学位17790个。

6.建成市县乡村四级互联互通的远程医疗服务体系，让基层群众享受更好的医疗健康服务。

7.完成中心城区36条主次干道架空线缆综合整治；继续实施中心城区背街小巷综合整治，今年改造100条，全面完成任务；用三年时间完成中心城区老旧小区改造，今年改造100个。

8.新建改建中心城区停车位3.2万个，其中公共停车位5000个；全部打通现有的16条“断头路”。

9.继续实施中心城区菜市场标准化改造升级，今年新建改造15个，全面完成任务。

10.中心城区新建绿道30公里，新增绿地面积100万平方米以上。

各位代表！新时代是奋斗者的时代。让我们更加紧密地团结在以习近平同志为核心的党中央周围，在省委省政府和市委的坚强领导下，只争朝夕，埋头苦干，不断开创新时代“一极两中心”建设新局面，以优异成绩迎接新中国成立70周年！
